# Wearable monitoring of sleep-disordered breathing: estimation of the apnea–hypopnea index using wrist-worn reflective photoplethysmography

**DOI:** 10.1038/s41598-020-69935-7

**Published:** 2020-08-11

**Authors:** Gabriele B. Papini, Pedro Fonseca, Merel M. van Gilst, Jan W. M. Bergmans, Rik Vullings, Sebastiaan Overeem

**Affiliations:** 1 Department of Electrical Engineering, Eindhoven University of Technology, 5612 AZ Eindhoven, The Netherlands; 2 Philips Research, High Tech Campus, 5656 AE Eindhoven, The Netherlands; 3 Sleep Medicine Centre Kempenhaeghe, 5591 VE Heeze, The Netherlands

**Keywords:** Health care, Population screening, Biomedical engineering

## Abstract

A large part of the worldwide population suffers from obstructive sleep apnea (OSA), a disorder impairing the restorative function of sleep and constituting a risk factor for several cardiovascular pathologies. The standard diagnostic metric to define OSA is the apnea–hypopnea index (AHI), typically obtained by manually annotating polysomnographic recordings. However, this clinical procedure cannot be employed for screening and for long-term monitoring of OSA due to its obtrusiveness and cost. Here, we propose an automatic unobtrusive AHI estimation method fully based on wrist-worn reflective photoplethysmography (rPPG), employing a deep learning model exploiting cardiorespiratory and sleep information extracted from the rPPG signal trained with 250 recordings. We tested our method with an independent set of 188 heterogeneously disordered clinical recordings and we found it estimates the AHI with a good agreement to the gold standard polysomnography reference (correlation = 0.61, estimation error = 3±10 events/h). The estimated AHI was shown to reliably assess OSA severity (weighted Cohen’s kappa = 0.51) and screen for OSA (ROC–AUC = 0.84/0.86/0.85 for mild/moderate/severe OSA). These findings suggest that wrist-worn rPPG measurements that can be implemented in wearables such as smartwatches, have the potential to complement standard OSA diagnostic techniques by allowing unobtrusive sleep and respiratory monitoring.

## Introduction

Obstructive sleep apnea (OSA) is one of the most common sleep disorders. It has an estimated global prevalence of 12% in adults, and is increasingly affecting the world population^1–4^. OSA is characterized by recurrent airflow reductions during sleep caused by complete or partial obstructions of the upper airway (i.e. obstructive apnea and hypopnea)^2^. OSA has both acute and chronic effects on health, such as daytime sleepiness and increased cardiovascular risk^2^. It is therefore paramount to timely diagnose and treat the condition. The gold standard technique to assess the presence and severity of OSA is polysomnography (PSG) in a sleep laboratory^5^. A certified sleep technician uses these measurements to annotate the sleep architecture and the presence of pathologically relevant sleep events, such as respiratory events, arousals and limb movements^6^. The ratio between the number of respiratory events and the total sleep time defines the apnea–hypopnea index (AHI). Although it has limitations^7,8^, the AHI remains the canonical metric to assess OSA severity as normal (AHI < 5 events/h), mild (5 $\le$ AHI < 15), moderate (15 $\le$ AHI < 30) and severe (AHI $\ge$ 30). Overnight recordings similar to PSG can also be performed at home using polygraphic home sleep apnea tests (HSAT), restricting the number of measured signals especially with regards to sleep measurement per se. Both PSG and polygraphic HSAT trade diagnostic accuracy with obtrusiveness. They are unsuitable, for instance, to perform population screening and to monitor OSA variability across multiple nights^3,9^.

Wearable devices could provide a level of unobtrusiveness unachievable with standard techniques, and as such enable faster OSA screening and improved long-term characterization and follow up. Especially wrist-worn sleep devices such as smartwatches or fitness trackers, are gaining attention from the sleep medicine community because of their promise to extend objective sleep monitoring over longer time periods in the home setting^10–12^. Most of these devices embed a green-light reflective photoplethysmography (rPPG) sensor plus a three-axial accelerometer. rPPG-based devices can extract cardiorespiratory parameters, such as heart rate variability (HRV) and surrogates of respiratory activity^13–15^. They have been shown to be able to assess sleep architecture in healthy and disordered populations^16–19^. As such, they constitute an attractive candidate for objective unobtrusive OSA monitoring.

In the last two decades, many studies were published on cardiovascular monitoring of OSA. Most of them focused on fingertip transmissive photoplethysmography and electrocardiography (ECG)^20^. Recent ECG-based methods showed good AHI estimation and OSA screening-performance in large and heterogeneous populations^21,22^. rPPG can potentially provide similar information as the ECG and it can be embedded in wrist-worn devices that are more accepted and easier to wear in a free-living context compared to ECG-patches or -belts^14,23^. However, the different physiological nature of these modalities may cause differences such as HRV mismatches^14,24^, hampering the direct application of algorithms developed for one sensor to another. A similar reasoning also applies to transmissive PPG-based methods^13,25,26^. In addition, most of the OSA monitoring methods using transmissive PPG employ the derived blood oxygen saturation measurement as input^20^; while the green light-rPPG usually embedded in wrist-worn devices cannot measure saturation^23^. Therefore, rPPG-based OSA monitoring approaches can be inspired by methods developed for ECG and transmissive PPG, but need to be re-validated and, most likely, adapted for this sensing modality.

The performance of cardiovascular-based OSA monitoring algorithms is influenced by the presence of other sleep disorders and associated events, and by the types of respiratory events^21,22,27^. Previously, we reported that other sleep disorders can constitute a confounding factor when detecting respiratory events^21,27^. For instance, limb movements tend to be mistaken for respiratory events, while hypopneas, the most common respiratory events, are often difficult to detect compared to apneas. The coexistence of such events especially complicates the cardiovascular-based monitoring of OSA, as the method has to balance sensitivity and precision to avoid underestimating or overestimating the AHI. Therefore, it is important to develop an AHI estimation algorithm using datasets that embrace the full complexity of healthy and disordered sleep.

Here, we propose a new AHI estimation method developed for wrist-worn, green-light rPPG devices, and asses its performance in a clinical population, comprising healthy subjects and patients with a various types and levels of disordered sleep. In addition to the HRV and movement features used in our previous ECG-based research^27^, we included respiratory activity features, sleep context information in the form of rPPG-based sleep stage probability and feature coverage. A deep learning model employed these features to detect 30-s epochs influenced by respiratory events (RE-epochs), and these RE-epochs were used to estimate the AHI. We tested our method on a heterogeneously sleep-disordered population of 252 recordings and we investigated the effect of enforcing a minimum rPPG quality (resulting in 188 recordings with reliable rPPG signal). Finally, we characterized our results based on clinical characteristics—e.g. medication intake and sleep characteristics—to explore factors affecting the AHI estimation and determine requirements for clinical implementation.

## Methods

### Datasets and split in training/validation/hold-out sets

We employed the SOMNIA and HealthBed datasets collected at the Kempenhaeghe Sleep Center^28^. We used the first 469 participants included in the SOMNIA database, which consists of simultaneous PSG recordings, clinical information and unobtrusive sensors recordings (e.g. the rPPG used for this research) collected during the standard diagnostic work-up of a heterogeneously sleep-disordered population. We combined this data with the first 33 participants belonging to the HealthBed database, a set of healthy adults without sleep disorders or other medical or psychiatric comorbidity, recorded with the same protocol as the SOMNIA database.

Table 1 reports the main characteristics of the population analyzed. The exclusion criteria for our combined datasets were sleep duration (detected using a rPPG-based algorithm) shorter than 30 min and use of continuous positive airway pressure (CPAP) during the PSG recording night. For this research, we used the signals from the wrist-worn rPPG device (32 Hz photoplethysmography and 128 Hz three-axial acceleration) as well as the modified lead II ECG signal (512 Hz). The wrist-worn device was developed by Philips for research purposes and has been used in several other biomedical research efforts, e.g. on blood pressure, sleep and atrial fibrillation monitoring^16,29,30^. Both datasets were manually annotated based on the full PSG by sleep technicians using the 2015 AASM guidelines. Importantly for OSA monitoring application, the presence of a hypopnea was defined by a reduction of airflow larger than 30% occurring together with an arousal or oxygen desaturation larger than 3%^31^. All technicians obtained the somnotechnologist rating from the European Sleep Research Society, and scoring proficiency was assessed in the inter-scorer reliability program of the American Academy of Sleep Medicine (https://isr.aasm.org/), yielding an overall agreement of 86 ± 8% and 97 ± 6% for respiratory and limb movement events. We used the manually scored clinical annotation (e.g. respiratory events and limb movements) and the clinical information (e.g. sleep onset latency, AHI and diagnosis). The OSA severity of each participant was determined according to the canonical AHI thresholds (in events/h): none with AHI < 5, mild with 5 $\le$ AHI < 15, moderate with 15 $\le$ AHI < 30, and severe with AHI $\ge$ 30.

We divided the 502 participants into three sets, i.e. training, validation and hold-out. The training and validation sets, with a 70–30% ratio, amounted to 250 participants (of which 229 from the SOMNIA dataset) with the recordings respecting two conditions (more details in “Features extraction”):

- less than 5% of the detected beats were suspected to be ectopic (based on the ECG signals)^32^,
- it was possible to calculate the features for at least 50% of the recording duration (for instance, features were not calculated when too many beats were missing).

The hold-out set consisted of 252 participants (of which 240 from the SOMNIA dataset) unselected with regard to the characteristics of the cardiovascular signal, i.e. no coverage or ectopic beats thresholds were imposed. The size of the hold-out set allowed for a representative number of participants in each OSA severity class, also taking into consideration the possible presence of comorbidities or other performance influencing factors.

All sets were sampled from the same pool of data (SOMNIA and HealthBed databases). The selection process adopted for training and validation sets allowed having high-quality data from which the deep learning model could learn the physiologically relevant information related to the presence of REs. Apart from a few minor selection criteria (no CPAP usage and a minimum sleep duration of 30 min), the hold-out set was the closest representation of the initial pool of data (SOMNIA and HealthBed databases) and therefore an adequate representation of the patient population visiting Sleep Medicine Center Kempenhaeghe.

The SOMNIA and HealthBed studies were reviewed by the medical ethical committee of the Maxima Medical Center (Eindhoven, the Netherlands. File no: N16.074 and W17.128). All participants provided written informed consent. All the studies met the ethical principles of the Declaration of Helsinki, the guidelines of Good Clinical Practice and the current legal requirements. The protocol for data analysis was approved by the Medical Ethical Committee of the Kempenhaeghe hospital (number 06.17) and by the Philips Institutional Review Board (Internal Committee on Biomedical Experiments, identification numbers ICBE-2-14791 and ICBE-2-18859).

**Table 1:** Demographics of the participants (and for each set).

	All participants	Training set	Validation set	Test set
Participants (#) (male)	502 (316)	175 (107)	75 (48)	252 (161)
Age (years)	48±16 [18–82]	49±16 [18–78]	48±15 [18–79]	48±16 [18–82]
BMI (kg/m^2^)	27±5 [15–49]	28±5 [15–42]	27±4 [17–40]	27±5 [15–49]
Total sleep time (min)	411±68 [102–580]	414±63 [199–580]	416±57 [243–561]	406±75 [102–540]
REM (% of sleep)	17±6 [0–35]	17±6 [0–32]	17±6 [5–31]	16±6 [0—35]
N1 (% of sleep)	14±7 [1–56]	13±7 [2–34]	13±7 [1–39]	15±8 [2–56]
N2 (% of sleep)	52±8 [30–93]	52±9 [32–93]	53±8 [33–73]	51±8 [30–78]
N3 (% of sleep)	17±9 [0–49]	17±9 [0–49]	16±8 [1–36]	17±9 [0–40]
Sleep efficiency (%)	82±13 [19–99]	83±11 [37–98]	83±9 [57–98]	81±14 [19–99]
AHI (events/h)	14±16 [0–91]	12±13 [0–70]	16±18 [0–71]	16±17 [0–91]
AI (events/h)	1±3 [0–31]	1±3 [0–24]	1±3 [0–20]	1±3 [0–31]
HI (events/h)	12±12 [0–86]	10±9 [0–47]	13±14 [0–64]	13±13 [0–86]
None/mild/moderate/severe OSA cases (#)	183/150/100/69	77/45/38/15	27/20/13/15	79/85/49/39
Participants with > 1 central apnea per hour (#)	68	24	16	28
Participants with > 1 mixed apnea per hour (#)	38	9	6	23
PLMI (events/h)	3±2 [0–19]	3±2 [0–9]	3±2 [0–9]	3±2 [0–19]
Disordered breathing/insomnia/movement disorder/parasomnia/no disorder cases (#)	241/121/57/35/70	93/45/17/13/20	44/13/8/4/6	104/63/27/18/44

Data area shown as mean ± standard deviation [range]. The OSA severity classes are none with AHI < 5, mild with 5 $\le$ AHI < 15, moderate with 15 $\le$ AHI < 30, and severe with AHI $\ge$ 30. Sleep efficiency is ratio of total sleep time to time in bed. AI and HI are respectively the number of obstructive apneas and hypopneas per hour of sleep. PLMI is periodic leg movement index calculated as number of periodic leg movements events per hour of sleep. The last row reports the five most common sleep disorder categories in the datasets (according to the primary diagnosis).

### Features extraction

We extracted features that describe the cardiovascular and the respiratory activity by analyzing the time and morphology characteristics of the rPPG pulses^15,33^. As a first step, the rPPG signal was segmented in pulses, and per pulse it was evaluated whether the quality of that pulse was sufficient through morphological comparison to a pulse template obtained from the one-hour portion of the rPPG signal containing that pulse^33^. The derived pulse quality index allows the removal of artefacts or pulses influenced by arrhythmic heart contractions which would affect the HRV features and the surrogate respiratory activity extraction^15,34^. The sinus rhythm pulses of good quality were then used to derive the inter-beat intervals (IBIs) necessary for the HRV analysis. Besides the rejection of pulses based on the pulse quality index, we removed an IBI and its preceding IBI when their ratio was larger than 1.5, due to the suspicion of being related to ectopic beats^27^. We derived the amplitude of each sinus rhythm pulse to extract a surrogate respiratory activity signal^15^ and, finally, from this surrogate determined the length and amplitude of each breath.

We used IBIs and breathing characteristics to calculate the HRV and respiratory activity features listed in Table 2. We also used the movement information provided by the three-axial accelerometer in the form of activity counts^15,16^. We calculated each HRV and respiratory feature over a feature-specific time window, and its value was associated with the central 30-s epoch within this window. This effectively leads to an epoch-by-epoch time resolution of the features. The HRV features are usually calculated over windows longer than the epoch definition. We calculated a shorter version of the HRV features to compensate for the smoothing effect caused by large calculation windows. We calculated the short HRV features only of the HRV features that would be still physiological representative and would allow a shortened calculation window. For instance, we excluded very low frequencies when performing the HRV frequency analysis on two minute windows and we did not calculate a shorter version of the detrended fluctuation analysis features. In addition to the cardiorespiratory features, we included the sleep stage probabilities—i.e. prediction probability of Wake, N1/N2, N3 and REM—and the feature coverage—i.e. the percentage of undefined features for each epoch due to lack of IBIs or respiratory activity coverage—obtaining a total of 212 features. A HRV feature was considered undefined at a certain epoch when the detected IBI covered less than half of the window used to calculate the feature. Similarly, a respiratory activity feature was considered undefined when less than three breaths were detected in the feature calculation window. We computed the sleep stage probabilities using the algorithm proposed by Fonseca et al.^16^. This algorithm employs a subset of the HRV features used in this research. Our method employed these additional sleep stage and coverage features to contextualize the HRV and respiratory features with respect to the different autonomic activity of each sleep stage and feature reliability. The Supplementary section Contribution of respiratory activity and sleep stage probability features reports the additional OSA monitoring value generated by including respiratory activity and sleep stages probability features as compared to a method focusing on HRV features only.

**Table 2:** Overview of the extracted features.

Features type	Number of features (number of those with window shorter than literature)	Described in	Features computation window sizes (s) (number of epochs for template comparison)
**HRV features**			
Arousal probability	10 (5)	^35^^#,&^	120^#^, 30^&^
Frequency analysis	23 (5)	^34^^#,&,^^36^^#,^^37^^#^	300^#^, 120^&^
Adapted frequency analysis	11 (5)	^38^^#,&^	300^#^, 120^&^
Detrended fluctuation analysis	5	^39–41^	360
Progressive detrended fluctuation analysis	1	^42^	60
Windowed detrended fluctuation analysis	1	^43^	360
High frequency pole analysis	4 (2)	^44^^#,&^	300^#^, 120^&^
Multi-scale entropy	20	^45–47^	540
Local phase coordination	7 (5)	^48^^#,^^49^^#,^^50^^&^	150^#^, 90^&^
Time analysis and statistics	74 (37)	^34^^#,&^	300^#^, 30^&^
Sample entropy	2 (1)	^51^^#,&^	300^#^, 30^&^
Visibility graph analysis	13	^52–55^	210
Hilbert transformation analysis	12 (6)	^56^^#,&^	300^#^, 30^&^
**Respiratory activity features**			
Amplitude analysis	5	^57^	150
Frequency analysis	3	^58^	30
Similarity respiratory patterns	2	^59^	30 (60)
Frequency peak analysis	3	^60^	30
Template distance	1	^58^	30 (50)
Time analysis	3	^61^	30
Visibility graph analysis	4	^52–55^	150
Variance	1	^60^	30
Sample entropy	1	^60^	180
**Accelerometer features**			
Activity counts	1	^29^	30
**Context features**			
Sleep stage probabilities	4	^16^	30
Features coverage	1	–	30

We calculated each feature using the methods proposed in the respective original methodological paper(s). Some of the features were calculated for different window sizes, and their number comprises both calculations (superscript symbols define the association between reference and the window size used).

We regularized the HRV, respiratory activity and activity count features by applying a Tukey-Ladder transformation followed by a z-score transformation^62^. The coefficients of these transformations were determined on the training set and applied to the validation and hold-out set. When the features were not defined, we set their value to zero, and these values were not used to compute the feature transformations and to train the model, i.e. loss weight was set to zero. The signals were automatically truncated based on the activity counts by removing periods with prolonged movements at the beginning and the end of the recording, as done by Radha et al.^29^, in order to automatically isolate the part in which the participant most-likely intended to sleep.

### The deep learning model for RE-epochs detection

Our deep learning model had the task of classifying 30-s epochs of each overnight recording as influenced by a respiratory event (RE-epochs, positive class) or not (non-RE epochs, negative class). Similar to our previous research, a 30-s epoch was labelled as RE-epoch if it includes at least 10 s of a respiratory event or if the beginning of an epoch is closer than 5 s to the ending of a respiratory event^27^. The model took all 212 features per epoch as inputs for the entire recording night (maximum 1150 epochs) of each participant, and its output was the probability of each epoch of being a positive class (values from 0 to 1). The probability threshold to label an epoch as positive class was derived based on the AHI estimation performance as explained in our previous ECG-based research^27^.

We trained over a thousand different models by combining different types of blocks of layers and hyper-parameters. Each model was trained with eight different participant randomizations of the training and validation sets, in order to assess its average performance. The randomized sets were the same for each model. We selected the final model, and the training and validation split, based on the AHI estimation performance. The Supplementary section Deep learning model reports the details regarding the training and model selection.

### AHI estimation

The AHI estimation was performed similarly to our previous ECG-based OSA monitoring research^27^. Our method estimated the AHI for each participant as the number of positively labelled epochs during sleep divided by the total sleep time. The same automatic sleep staging algorithm used to calculate the sleep stages probability provided the sleep and wake classification and, consequently, the total sleep time^16^. Our method corrected the AHI obtained by this ratio by a multiplicative coefficient derived by linearly regressing the reference AHI obtained from the manual annotations with the AHI calculated from the number of positive class reference epochs. Differently from our previous research, we excluded from the AHI estimation the epochs with more than 80% of undefined features to avoid biasing the AHI estimation with less reliable epochs.

### Analysis

#### Hold-out set selection based on rPPG quality

The quality of the rPPG can influence the features extraction and, consequently, the performance of our method. Therefore, we decided to threshold several rPPG quality parameters in order to ensure the reliability of the hold-out set recordings. The selected rPPG quality parameters and their thresholds are reported in in Table 3. While during features extraction, the pulse quality index was used to remove single pulses, here it was used together with the IBI coverage to assess the overall quality of each rPPG recording. The thresholds to isolate low quality recordings were calculated as the tenth percentile of the averages per recording of in the training set. The selected version of the hold-out set consisting of 188 participants (of which 180 from the SOMNIA dataset) was isolated to demonstrate that it is possible to improve the AHI estimation reliability by enforcing a minimum level of rPPG quality. We report the main results of our method for both the complete hold-out set and the hold-out without low rPPG quality recordings, but we focus part of the results analysis only on the latter.

**Table 3:** rPPG quality recording exclusion criteria.

rPPG quality metrics	Minimum values
IBI coverage (%)	83
Average pulse quality index	0.85
Median pulse quality index	0.90
Percentage of pulses with quality index > 0.6 (%)	89
25th percentile pulse quality index	0.80
75th percentile pulse quality index	0.96

These metrics were calculated for each 30-s epoch and averaged for each recording. We considered the recordings not satisfying one or more of the minimum values characterized by low rPPG quality.

#### RE-epoch detection

The RE-epoch detection performance was analyzed for the hold-out set (with and without the recording exclusion based on rPPG quality) by calculating Cohen’s kappa, accuracy, sensitivity, specificity, and positive predictive value (PPV) between the reference and the detected RE-epochs. In addition, we report Cohen’s kappa maximum along with the prevalence and bias indexes in order to contextualize the Cohen’s kappa values, as suggested by Sim and Wright^63^. The area under the curve of PPV-sensitivity (PR AUC) and receiver operating characteristics (ROC AUC) plots were also calculated between the reference RE-epoch and the output probability of deep learning model, i.e. before applying the probability threshold. We calculated these metrics on all epochs contributing to the AHI estimation.

We investigated the effect of different sleep events on the true- and false-positive detection. Regarding the effect of different respiratory events on the performance, we calculated the sensitivity for RE-epochs containing at least one respiratory event (in case more than one event was present, the longest one determined the respiratory event label of the epoch). Regarding the presence of limb movement events, we investigated the sensitivity, specificity and PPV concerning epochs during which such events occurred and compared them to the sensitivity, specificity and PPV for the rest of the epochs. We focused on limb movements because they are known to cause false-positive detection of respiratory events but also to occur at the end of respiratory events^27^. The epochs were associated with these events if they were the longest event in the epoch, and their duration was longer than 3 s.

#### AHI and OSA severity estimation

We analyzed the AHI estimation performance graphically using Bland–Altman and linear regression plots. As a measure of the correlation between estimated and reference AHI, we employed the Spearman’s correlation instead of the Pearson’s correlation because the data were heteroscedastically related, i.e. the variability of the estimated AHI was unequal with respect to the reference AHI values (p < 0.01 with Breusch–Pagan test^64^). Besides, we calculated the intraclass correlation coefficient (ICC) to provide an indication of the inter-rater variability between the “algorithm scorer” (estimated AHI) and the human scorers (reference AHI). We opted for an ICC(2,1) measuring absolute agreement according to guidelines given by Koo et al.^65^. We exploited ROC curves to graphically represent the screening performance for the canonical AHI thresholds when varying the screening threshold applied to the estimated AHI. Also, we investigated sensitivity, specificity, accuracy and Cohen’s kappa for each screening canonical threshold. For screening performance, Cohen’s kappa was calculated, reporting the maximum, prevalence and bias indexes^63^. The OSA severity estimation was shown using confusion matrixes and linearly weighted Cohen’s kappa^66^, to account for the ordinal nature of the OSA severity scorings (reported together with the corresponding kappa maximum^63^).

#### Participant characteristics influencing the AHI estimation performance

The participants’ characteristics can influence the AHI estimation performance. For instance, age affects both the HRV and the sleep architecture, two key parameters in our method^67,68^. However, most of the participants’ characteristics are not independent of each other, or they might have a combined effect on the performance. Therefore, we investigated which characteristics influenced the AHI estimation error, i.e. reference minus estimated AHI, by means of an elastic net^69^. This method consists of linear regression with lasso and ridge penalization that sets to zero the coefficient of independent variables (participants’ characteristics) that do not contribute in explaining the dependent variable (AHI estimation error). The regression was performed on the hold-out dataset without low-quality rPPG recordings to highlight the contribution of sleep and physiological characteristics rather than the quality of the recordings. The amount of regularization and the proportion between lasso and ridge penalization were estimated using a fivefold cross-validation on the investigated data. The participant and recording characteristics that were included as independent variables are: rPPG quality metrics, age, sex, sleep onset latency, wake after sleep onset, total sleep and recording times, time spent in each sleep stage and per-hour of sleep, absolute amount of awakenings and per-hour of sleep, average and standard deviation of time spent per sleep cycle in REM and N3, number of sleep cycles, absolute number of sleep stage transitions and per-hour of sleep, and percentage of ectopic beats (based on the ECG signal, as defined in section “Features extraction”). A z-score normalization was applied to the regression variables before the fitting.

We also investigated cases with substantial AHI underestimation and overestimation, defined as:

1 $$\begin{aligned} \textit{Considerable AHI underestimation}&=\left\{ \begin{array}{lcl} estimated~AHI< \dfrac{1}{2}\times AHI - 2.5, &{} 5\le AHI<15 \\ estimated~AHI < \dfrac{2}{3} \times AHI - 5, &{} AHI\ge 15 \\ \end{array}\right. \end{aligned}$$

2 $$\begin{aligned} \textit{Considerable AHI overestimation}&=\left\{ \begin{array}{lcl} estimated~AHI> 2\times AHI + 5, &{} 0\le AHI<15 \\ estimated~AHI >\dfrac{3}{2} \times AHI + 7.5, &{} AHI\ge 15 \\ \end{array}\right. \end{aligned}$$

With respect to the measure based on the limits of agreement used in our previous paper^27^, the criterion employed here allowed the identification of cases in which the estimated or the reference AHI were low but, in proportion, still considerably different from each other. For these participants, we further investigated the clinical picture and recording characteristics, e.g. the presence of other comorbidities, obstructive apnea and hypopnea indexes (AI and HI), medication influencing cardiac activity, and severity of oxygen desaturations.

## Results

### RE-epoch detection performance

**Figure 1:**
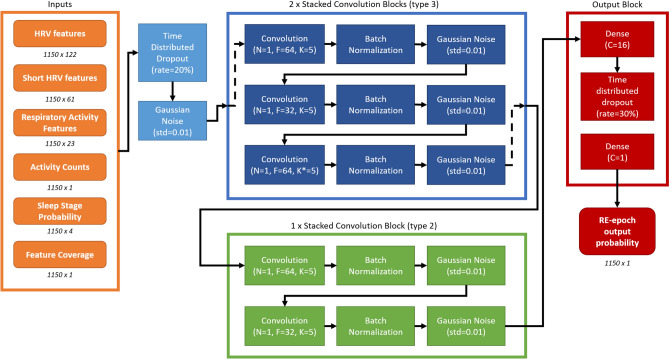
The selected model architecture for the RE-epoch detection. The numbers below boxes indicates the dimensions (with 1150 being the maximum number of epochs). The *rate* indicates the drop out rate, *N* indicates the number of stacked convolution, F the number of filter, *K* the kernel size, K* the kernel size with dilation rate of 2, *C* the number of units of the dense layers and std the standard deviation of the Gaussian noise. The block types are further described in the Supplementary section *Deep learning model*.

Figure 1 reports architecture and hyper-parameters of the selected deep learning model. This model completed its training in 75 training iterations and it had a Spearman’s correlation of 0.77 with p < 0.01 and an OSA severity Cohen’s kappa of 0.37 in the validation set.

Table 4 reports the RE-epoch detection performance of the best model on the hold-out set for the output probability threshold of 0.65 obtained from the training set.

To exclude the sleep staging algorithm developed by Fonseca et al.^16^ as a possible source of error, we checked its performance on our hold-out set. The sleep staging algorithm had a good agreement with the reference hypnogram (manually scored PSG) and the results were not different from the original publication (four-class sleep scoring Cohen’s kappa: 0.56, and sleep/wake classification Cohen’s kappa: 0.62)^16^.

**Table 4:** RE-epoch detection performance on the hold-out set with and without the low rPPG quality recordings exclusion.

Recordings	Number of epochs (RE-epochs) (#)	Prevalence /Bias index^63^	Cohen’s kappa (kappa max^63^)	Accuracy (%)	Sensitivity (%)	Specificity (%)	PPV (%)	PR AUC	ROC AUC
All	222039 (32456)	0.73/0.04	0.36 (0.80)	85	38	94	53	0.48	0.80
Low rPPG quality recordings excluded	171237 (22305)	0.75/0.03	0.37 (0.84)	86	39	94	51	0.47	0.82

The performance is for the overall amount of epochs contributing to the AHI (i.e. not Wake and epochs with less than 80% undefined features).

The sensitivity of the detection of RE-epochs changed depending on the type of predominant respiratory event they included, with the hypopneas being the type of respiratory event with the lowest sensitivity (Table 5). The 42% of the false-positive detections consisted of epochs characterized by limb movements (40% with the exclusion of low-quality rPPG recordings). The false-positive detections characterized by limb movements amounted to 11% of the total number of epochs characterized by these movements (same with the exclusion of low-quality rPPG recordings). The detection of RE-epochs coinciding with limb movements had a lower specificity and PPV, and higher sensitivity than epochs without limb movement events (Table 6).

**Table 5:** RE-epoch detection performance in the hold-out set with and without the low rPPG quality recordings.

Recordings	Respiratory event	Number of RE-epochs (% with respect to total RE-epochs)	Sensitivity (%)
All	Hypopnea	23371 (78)	34
	Obstructive apnea	2315 (8)	61
	Mixed apnea	1251 (4)	84
	Central apnea	1222 (4)	40
Low rPPG quality recordings excluded	Hypopnea	16909 (82)	37
	Obstructive apnea	1544 (7)	60
	Mixed apnea	382 (2)	78
	Central apnea	572 (3)	60

The performance is for the overall number of epochs contributing to the AHI (i.e. not Wake and epochs with less than 80% undefined features).

**Table 6:** RE-epoch performance for epochs characterized or not by limb movement events in the hold-out set with and without the low rPPG quality recordings.

Recordings	Sleep event	Number of epochs (% of RE-epochs)	Sensitivity (%)	Specificity (%)	PPV (%)
All	Not limb movements	126219 (10)	38	96	67
	Limb movements	39667 (2)	38	88	32
Low rPPG quality recordings excluded	Not limb movements	100021 (9)	39	96	65
	Limb movements	28428 (2)	43	88	33

Only the epochs contributing to the AHI calculation are taken into consideration (i.e. not Wake and epochs with less than 80% undefined features).

### AHI estimation

**Figure 2:**
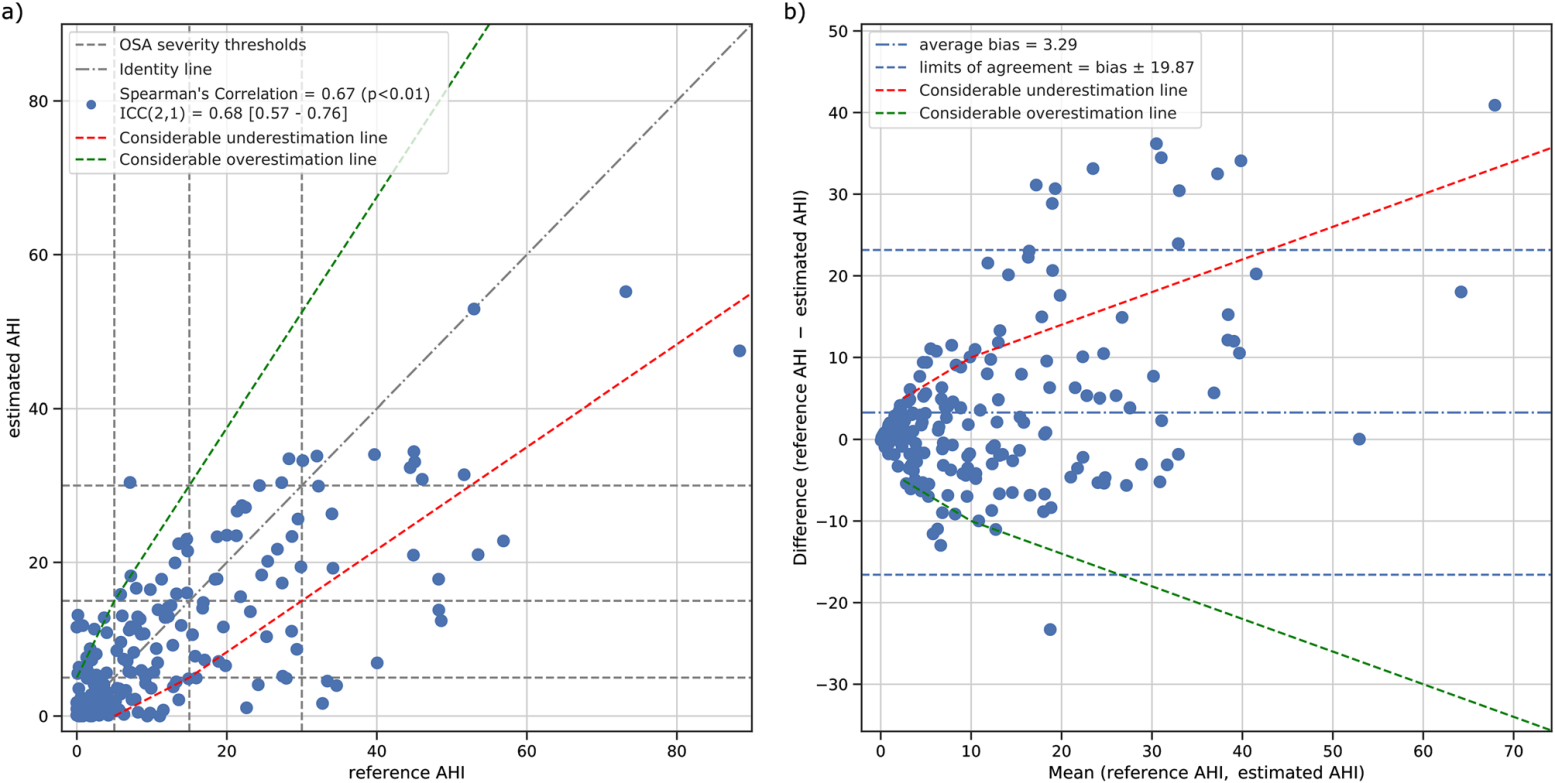
Analysis of the estimated AHI performance after removal of low-quality rPPG recordings. (**a**) Correlation between reference AHI versus estimated AHI; dashed lines delimit the canonical OSA severity classes and the dash-dotted line is the identity line. (**b**) Bland–Altman plot of the reference AHI and estimated AHI. The bias and the limits of agreement (i.e. 1.96 times the standard deviation of the difference) are shown as events/h. The red and the green dashed lines represent, respectively, the boundaries to define considerable under- and overestimations.

The estimated AHI significantly correlated with the reference AHI obtained by manual scoring of the recordings. Prior to excluding the recording with low rPPG quality, Spearman’s correlation between the reference and estimated AHI was 0.61 (p < 0.01) and the ICC(2,1) was 0.64 with a 95% confidence interval of [0.51–0.74]. The average bias and limits of agreement of the AHI estimation for the entire hold-out set were 4.7 ± 23.5 events/h. After the exclusion of recordings with low rPPG quality, the Spearman’s correlation further increased to 0.67 (p < 0.01), while the ICC(2,1) increased to 0.68 (95% CI [0.57–0.76]). The average bias and limits of agreement for the high rPPG quality hold-out set were 3.3 ± 19.9 events/h. Figure 2 gives a graphical overview of the results for the AHI estimation for the hold-out sets after the exclusion of recordings with low rPPG quality.

**Figure 3:**
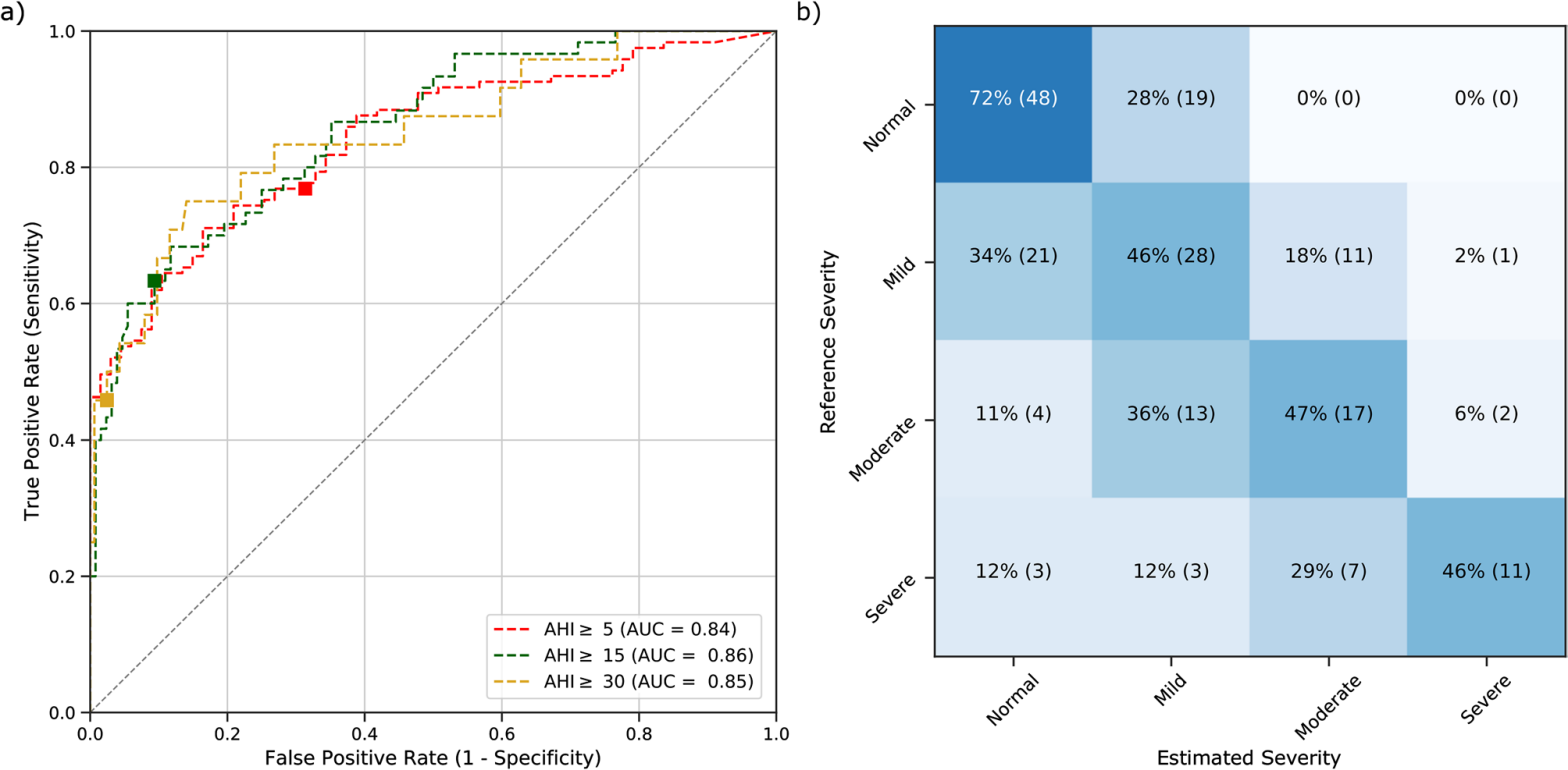
Receiver operating characteristics and confusion matrix of the estimated for the three canonical AHI thresholds after removal of low-quality rPPG recordings. (**a**) AUC area under each curve; square markers indicate the points in the curve where the estimated AHI threshold for severity classification is equal to the canonical 5, 15 and 30 events/h. (**b**) OSA severity classes obtained from the AHI (reference severity) and estimated AHI (predicted severity) using the canonical thresholds. In each cell, the percentage per severity is shown (also visually indicated by the color scale) as well as the number of participants.

Table 7 groups the screening performance of our estimated AHI. Similarly to the AHI estimation, removing recordings with low rPPG quality increased the screening performance. The weighted Cohen’s kappa between reference and estimated OSA severity was 0.46 (maximum 0.77) and 0.51 (maximum 0.85) respectively with and without the low rPPG quality recordings. Figure 3 shows the ROC curves for the three canonical AHI screening thresholds and the confusion matrix of the OSA severity classes.

**Table 7:** Screening performance for the estimated AHI with respect to the reference AHI for the canonical screening thresholds for the hold-out set with and without the low rPPG quality recordings.

Recordings	Screening threshold	Participants above the threshold (#)	Sensitivity (%)	Specificity (%)	PPV (%)	Prevalence/Bias index^63^	Cohen’s kappa (kappa max)^63^	ROC AUC
All	AHI $\ge$ 5	173	72	71	84	0.27/0.10	0.39 (0.78)	0.80
	AHI $\ge$ 15	88	59	90	75	0.38/0.07	0.51 (0.82)	0.82
	AHI $\ge$ 30	39	41	98	80	0.77/0.07	0.49 (0.64)	0.84
Low rPPG quality recordings excluded	AHI $\ge$ 5	121	77	72	83	0.24/0.05	0.47 (0.90)	0.84
	AHI $\ge$ 15	60	62	91	75	0.42/0.06	0.55 (0.86)	0.86
	AHI $\ge$ 30	24	46	98	79	0.80/0.05	0.53 (0.71)	0.85

### Factors influencing the AHI estimation performance

We found the AHI estimation error to be explained by the percentage of sleep spent in N3 (linear coefficient=0.10), the age ([years], − 0.12), the sleep onset latency ([min], − 0.03), the total recording time ([min], − 0.02), the reference AHI ([events/h], 0.54), and a constant value (11.66). The linear regression was able to explain 55% of the total variance ($r^2$ = 0.55). The regression statistics showed that only reference AHI and age were significant (p < 0.05).

Considerable overestimation only occurred in 5% of participants (9 participants, 7 females). Most had disorders other than OSA: parasomnias (n = 2), sleep related movement disorders (n = 2), chronic fatigue syndrome (n = 2), and one with sleep-related abnormal swallowing. Eight of these had no OSA, but were estimated to have mild OSA. The participant with the abnormal swallowing was estimated as severe OSA while suffering from mild OSA according to the gold standard (with an AHI of 7.1 events/h).

**Figure 4:**
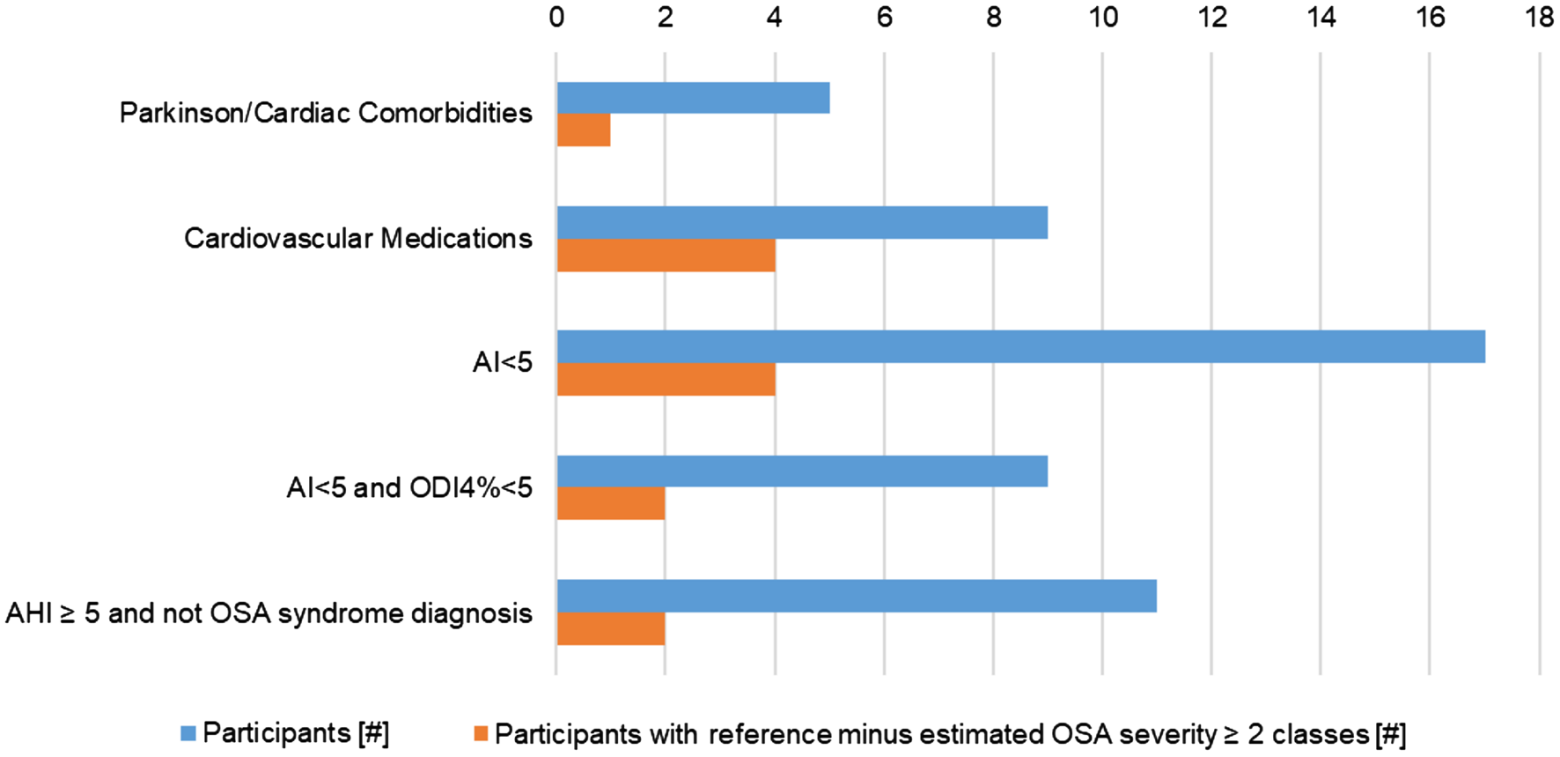
Characteristics of the considerable underestimated participants that might have influenced the underestimation (for all the participants and for those with at least two class difference between reference and estimated OSA severity). Cardiac comorbidities include bundle-branch-block, premature ventricular/atrial contraction and paroxysmal atrial fibrillation. Cardiovascular medications include anti-arrhythmic compound, ACE-inhibiters, beta-blockers and thyroid hormones.

Considerable underestimation of the AHI occurred more frequently, in 30 participants (16% of participants; 11 females) using our definition. Importantly, OSA classification was only limitedly affected, with seven having an estimated OSA severity two classes lower than the reference (e.g. severe OSA with a mild OSA estimation), and three a difference of three OSA severity classes. Figure 4 shows the distribution of factors that might have influenced the accuracy of our method. In the large majority of participants with underestimation of the AHI, a (clinical) explanation for the discrepancy was found, with only five cases where no reason could be identified.

The number of zero-weight epochs, i.e. epochs with less than 80% defined features, influences the AHI estimation performance because it reduces the amount of epochs contributing to the AHI. To contextualize the results regarding these epochs, we calculated the per-recording percentage of zero-weight epochs with respect to the total number of epochs. The hold-out set after the exclusion of low rPPG quality recordings had 0% [0–3%] (median [interquartile range, IQR]) zero-weight epochs. The excluded recordings had 5% [1–31%] zero-weight epochs. The two groups did not have a normal distribution (p < 0.01, Shapiro–Wilk test^70^), and were statistically different (p <0 .01, Mann–Whitney test^71^).

We investigated the effect of tightening the rPPG quality requirements on performance. The Supplementary section *Influence of rPPG quality on the performance* summarizes these results. Overall, the stricter the quality requirement, the better the performance. However, the higher quality standard significantly reduced the number of recordings included in the analysis, and reducing the results’ interpretability as a result. In the main results, we therefore opted for lower recording quality requirements to provide a more generalizable overview of our method performance.

## Discussion

We developed and tested a method to estimate the AHI using reflective PPG which can be implemented on devices such as smartwatches and fitness trackers. The AHI obtained with our method allows OSA screening and OSA severity estimation, even in a heterogeneously sleep-disordered population with a high likelihood of cardiovascular confounding factors and a large percentage of hypopneas.

The estimated OSA severity showed a fair to good agreement with the reference^72^ and was also in line with automatic AHI estimation in HSAT, one of the current gold standards. As an example, we achieved a similar distribution in the OSA severity class and similar underestimation tendency of the two HSATs with automatic AHI estimation investigated by Aurora et al.^73^. In comparison with those, our method presented a higher amount of overestimated severity cases. However, this could be expected given that cardiovascular information used by our method is more prone to false positives than the signal usually measured with HSAT (e.g. respiratory signals and oxygen saturation), especially in a heterogeneously sleep-disordered population like ours.

The agreement between reference and estimated AHI, quantified by the ICC, was good to moderate^65^ and, after applying the rPPG quality inclusion criteria, it was comparable with automatic AHI estimation with HSAT. For instance, Malhotra et al. reported an ICC with a 95% confidence interval of 0.91 [0.58–0.97] for automatic AHI estimation with an HSAT-dedicated algorithm^74^. However, they obtained this result with HSAT-recordable signals, using the AASM 2007 scoring rules with alternative hypopnea criteria (> 50% respiratory amplitude with > 3% oxygen saturation or arousal)^31^, the average of 10 human scorers as reference AHI, and a population of 70 good quality recordings without the inclusion of other disorders, such as insomnia or cardiac comorbidities^74^. Being consistently above their lowest confidence interval boundary represents a significant result for our method and the different experimental set-up might have largely contributed to the performance difference.

Our AHI estimation method has the potential to be used as an OSA screening tool. The tendency of our method to underestimate the AHI translated in a lower screening sensitivity for moderate and severe cases. However, the high ROC–AUC values allow for compensating for this tendency by lowering the screening threshold used for the moderate and severe cases, trading in specificity for sensitivity. Lowering the threshold for severe cases from the canonical 30 to 20 events/h would increase sensitivity with 21% (from 46 to 67%) and decrease specificity with 8% (from 98 to 90%). Lowering the threshold for moderate cases from 15 to 10 events/h would increase sensitivity with 15% (from 62 to 77%) and decrease specificity with 16% (from 91 to 75%).

In light of the results obtained, there are several target possible applications/populations for our method, according to different OSA monitoring goals. The first is the general population, in which the approach could be employed as a screening tool, especially when other sleep disorders are present or suspected. In this scenario the device might be used during multiple nights to increase screening sensitivity and reduce the influence of low-quality recordings. The second could be a population with an ambiguous OSA profile, e.g. known mild/moderate OSA but not fully fitting symptomatology. In this case our method might be used for confirmation of the PSG-based diagnosis in a home monitoring context. Finally, the method lends itself well for treatment follow up, for example detection of exacerbation of OSA over time, or occurrence of comorbid sleep disorders such as insomnia.

Interestingly, the RE-epoch detection and AHI estimation performance further improved compared to our ECG-based method^27^. In that study we achieved, for a subset of the SOMNIA and HealthBed datasets of this research, a Spearman’s correlation for AHI estimation of 0.50 with a bias and 95% limits of agreement of − 0.51 [− 25.8 to 24.79] events/h. The current method achieved higher performance than the ECG-based method even with unfavorable premises, such as rPPG being significantly more prone to missing or unreliable features due to artefacts^14,75^. The performance increase is likely due to the more complex RE-epoch detection model used along with the additional features employed.

The AHI estimation performance might appear to contrast with the RE-epoch detection performance with respect to the low sensitivity and Cohen’s kappa. Relying on the OSA severity classification performance to choose the best model and tune the output probability threshold penalized the sensitivity to favor the correct classification of participants without OSA in the presence of confounding factors, such as other sleep disorders. This behavior was intended in order to provide accurate AHI and OSA severity estimations rather than an accurate RE-epoch detection, since the former are the clinically relevant parameters. However, the overall structure of our method remains valid also in case a high RE-epoch detection performance would be preferred. As an example, for the hold-out set without low-quality recordings, choosing the output probability threshold based on the per-recording f1 score, would have increased sensitivity to 60% (+ 31% with respect to Table 5) and decreased specificity to 84% (− 10% with respect to Table 5), but also significantly lowered the OSA severity weighted Cohen’s kappa from 0.51 to 0.32. Future work will focus on improving the epoch-by-epoch detection without sacrificing AHI estimation accuracy in order to provide additional OSA-related information.

Artefacts, noise and arrhythmic beats affecting the rPPG quality could potentially weaken the link between the extracted cardiovascular activity and presence or absence of respiratory events. However, excluding recordings with low rPPG quality did not yield a definite improvement on the RE-epoch detection performance. Probably, increased rPPG reliability was overshadowed by the simultaneous increase of sensitivity to hypopneas and increased prevalence of this type of events (due to the exclusion of part of the recordings). The exclusion of low-quality recordings influenced the sensitivity to mixed apneas and central apneas as well, but the low number of epochs precluded any solid physiological hypotheses. For AHI estimation performance, there was a clear positive effect of rPPG quality-based exclusion. The higher amount of zero-weight epochs for the recordings with low rPPG quality entailed a lower amount of epochs contributing to AHI estimation and increased the chances of underestimating the AHI, especially considering that our method is very robust but only moderately sensitive (high specificity and low sensitivity in detecting RE-epochs). Therefore, removing low-quality recordings reduced the underestimation and consequently increased AHI-related performance.

The mechanisms leading to low quality rPPG recordings can be categorized as physiological or technical. The first category encompasses phenomena that predominantly determine pulse timing and morphology over the occurrence of REs (e.g. arrhythmias). The detrimental effect of these phenomena cannot be directly corrected for, but might be circumvented by developing features retaining more sleep-related information despite their presence. The second category encompasses factors that disrupt the rPPG signal and impede its capability to describe local blood volume variations (e.g. movements or not optimal skin contact). These phenomena might be corrected, for instance by changing the sensor placement to less movement-susceptible body areas. These measures may reduce recording exclusion due to low quality and as a result increase performance.

We chose to have a considerably larger hold-out set compared to the training set. Decreasing the hold-out set (in favor of the training set) would risk compromising the presence of severe OSA cases in the hold-out set and limit the possibility to draw conclusions for this group. We tried to retrain the selected deep learning model with additional data (100 recordings coming from the SOMNIA database and with similar characteristics of the datasets used) and decrease the validation size in favor of the training size. However, this did not lead to any significant performance increase. Nevertheless, we think that the addition of new training data may improve performance, but only if the data are carefully selected to increase the number of participants with specific characteristics that are easily misclassified. This will be a topic of future research, when such data becomes available.

The amount of considerable overestimation was small for the large size and complex variety of the population tested. The overall resilience of our method to AHI overestimation may even be better in everyday applications. Most of these overestimated cases presented pathologies known to act as cardiovascular confounding factors, such as sleep related movements^27,76,77^. In case of OSA screening, overestimating participants with sleep disorders other than OSA has minor consequences since these participants should anyway be referred to a sleep clinic to assess their condition.

Considerable AHI underestimations might be more critical for the application of our method as an OSA screening tool because they might fail to trigger standard sleep investigations. Fortunately, most of the underestimated cases in our study consisted of OSA severity misclassification of only one class; and the presence of OSA was flagged in most of the cases with moderate or severe OSA. Besides, the estimated AHI of several cases (10 out of 30) was more in line with the clinical diagnosis than the reference OSA severity classification: our method assigned a normal OSA severity to participants with an AHI > 5 events/h but without associated clinical symptoms. This indicates that these underestimations were of limited clinical relevance and have minor impact on the potential of our method as a screening tool.

In addition, 25 out of the 30 underestimated cases could be explained by factors influencing the cardiovascular system or specific sleep related characteristics. Several were characterized by disorders and/or medications directly affecting autonomic nervous system activity, heart contractility, vascular tone or blood pressure regulation^34,78–81^. These factors likely influence cardiovascular responses to respiratory events and decrease the reliability of the features used by our method. Most of these factors can be easily screened during clinical intake and may trigger the use of standard diagnostic techniques such as PSG, instead of cardiovascular-based monitoring. Paroxysmal disorders, like some arrhythmias, might be unknown to the participants however, and future work will therefore focus on developing rPPG-based solutions to isolate those cases to prevent AHI estimation errors.

Several underestimated participants presented principally hypopnea events with low desaturation values, i.e. AI and 4% oxygen desaturation index lower than 5 events/h. This underestimation is in line with the literature based on PSG and HSATs: hypopnea events, and especially those with a desaturation lower than 4%, are a source of higher disagreement among human and automatic scorers^73,82^. Our work remarks the controversy regarding the hypopnea definition and its relation with clinical outcomes^31,83^, at the point that it is questionable to consider as underestimated participants those not diagnosed with OSA syndrome (i.e. mild OSA plus symptoms) with a reference AHI > 5 and estimated AHI < 5.

To investigate factors leading to underestimation, we looked into the recordings with the highest OSA severity misclassification, namely the three severe OSA participants misclassified as normal. These participants had an AHI between 30 and 35 events/h, so were in fact close to moderate OSA. One participant has mostly hypopnea (AI = 0.2), and used cardiovascular medication. Another participant had only 3 hours of sleep (37% sleep efficiency) while the sleep staging algorithm reported 7 h of sleep. Therefore, the estimated AHI was influenced by the low agreement between the real and the estimated sleep duration. The third participant did not have clear factors that might have influenced AHI estimation. For this participant, the output probability of the RE-epoch and not-RE-epoch were significant difference between (Mann–Whitney test^71^, p < 0.01); however, the RE-epoch output probability values were mostly below the threshold (median and IQR: RE-epoch 0.24 [0.14–0.47], not-RE-epoch 0.16 [0.10–0.27]). This indicates that the detection algorithm was able to capture the effect of the REs, but it could not confidently label them as RE-epochs. This uncertainty might be corrected by increasing the number and the variety of severe OSA participants during the training of the deep learning model.

The AHI error was found to be significantly explained by the reference AHI and age. The effect of the AHI was expected due to the found underestimation tendency combined with the lower-bounded definition of this quantity, i.e. AHI cannot go below zero. The overestimating effect of age might be due to the combination of reduced sleep time, reduced slow wave sleep, and an increase in arousals that may lead to a decrease of the AHI denominator, a reduced presence of the stage with lowest likelihood of respiratory events, and an increased chance of false-positives^67^, respectively. Besides, the increase of sympathetic activity with age might have increased the false positive detection due to sympathetic activations being characteristic for respiratory events^67,84^. The effect of age and reference AHI on the estimation error might be decreased by enlarging the training set with older participants and more severe OSA cases. Besides, developing features independent of the age might also help in reducing this detrimental effect.

As previously mentioned, there might be disagreement among human scorers regarding the presence of REs and, consequently, the AHI. Having multiple scorers for each recording would have mitigated this issue, but was not available in this case. This is a limitation of our study, although all scorers were from a small group of experienced dedicated sleep technicians, with certified diagnostic performance in the AASM inter-rater variability program. Nevertheless, we feel that that the large number of recordings, the heterogeneity of the data and the usage of multiple scorers allowed fair assessment of method performance.

Although our method was developed for rPPG recordings, it may be applicable to other sensing modalities. We employed features that are sensor agnostic; for instance, respiratory activity and an activity count surrogate can be extracted from the ECG signals^24,85^. In addition, several sleep monitoring methods have been described which were developed for one sensing modality but could be applied to other modalities directly or with some adaptations. For example, Fonseca et al.^16^ proposed a sleep staging algorithm trained on ECG data that had similar performance on rPPG data (if taken in account the higher likelihood of noise and artefacts in rPPG signals). Phillips et al.^86^ trained a deep learning model for OSA monitoring using ECG data and applied a domain adaptation technique to obtain a model for rPPG data without need of a large rPPG dataset. Similar approaches could be applied to our method and we aim to investigate this in future work to focus on the physiological and technical differences between sensing modalities.

Recently released wrist-worn consumer devices embed red and infra-red rPPG, in addition to the most common green-light rPPG, enabling the measurement of relative changes in oxygen saturation. The sensitivity and specificity of these sensors have yet to be clinically confirmed. If proven to be accurate for low oxygen desaturation values as well, these techniques will be a very useful addition to cardiovascular-based OSA monitoring methods such as ours.

## Conclusion

We described an AHI estimation method based exclusively on information retrieved from wrist-worn rPPG devices. We tested our method in a heterogeneous clinical sleep population and investigated in detail the characteristics of our estimated AHI in comparison with the reference AHI obtained by expert human scorers. We found that the proposed rPPG method might be employed as an OSA monitoring tool.

Although rPPG devices cannot fully substitute PSG and HSAT due to the lower amount and more indirect nature of physiological information extracted, future development of our approach might complement these standard techniques by allowing sleep to be monitored continuously for long periods at home in an unobtrusive way. These characteristics could open new scenarios for OSA monitoring. For instance, wrist-worn PPG devices could add an objective observation to subjective screening of OSA with questionnaires, quantify the night-to-night variability of the disorder, combine night-time and daytime monitoring, be used to follow up on treatment and provide a low-cost objective solution for large-scale screening^3,10–12,87^.

## Supplementary information

Supplementary Information.

## Data Availability

The data used in this study are available from the Sleep Medicine Centre Kempenhaeghe upon reasonable request. The data can be requested by presenting a scientific research question and by fulfilling all the regulations concerning the sharing of the human data (e.g. privacy regulations). The details of the agreement will depend on the purpose of the data request and the entity that is requesting the data (e.g. research institute or corporate). Each request will be evaluated by the Kempenhaeghe Research Board and, depending on the request, approval from independent medical ethical committee might be required. Specific restrictions apply to the availability of the data collected with sensors not comprised in the standard PSG set-up, since these sensors are used under license and are not publicly available. These data are however available from the authors upon reasonable request and with permission of the licensors.
