# Supplementary material for: Wearable monitoring of sleep-disordered breathing: estimation of the apnea–hypopnea index using wrist-worn reflective photoplethysmography
Source: Sci Rep. 2020 Aug 11;10:13512. doi: 10.1038/s41598-020-69935-7 (PMC7421543; doi:10.1038/s41598-020-69935-7)
Supplement: Supplementary file 1 — Supplementary Information. [file 41598_2020_69935_MOESM1_ESM.pdf]

Supplementary information: Wearable monitoring of sleep-disordered breathing: estimation of the apnea-hypopnea index using wrist-worn reflective photoplethysmography

Gabriele B. Papini[1,2,3,*], **Pedro Fonseca**[1,2], **Merel M. van Gilst**[1,3], **Jan W.M. Bergmans**[1,2], **Rik Vullings**[1], **Sebastiaan Overeem**[1,3]

[1] Eindhoven University of Technology, Dept. of Electrical Engineering, Eindhoven, 5612 AZ, The Netherlands
[2] Philips Research, High Tech Campus, Eindhoven, 5656 AE, The Netherlands
[3] Sleep Medicine Centre Kempenhaeghe, Heeze, 5591 VE, The Netherlands
[*] g.p.papini@tue.nl

Contribution of respiratory activity and sleep stage probability features

To the best of our knowledge, our method was the first one using respiratory activity features and sleep stages probability as input features for OSA monitoring tasks with rPPG devices. Therefore, we proved the additional values of these features by developing a method focused on HRV, similar to the one we previously published for ECG signal[27]. This method employed only HRV features, activity counts and feature coverage (HRV-centered method) as input for a deep learning model. The model was selected based on the models provided to step 2 of the deep learning model selection procedure (section Deep learning model). Starting from step 2 allowed having a model tuned for the reduced feature set without repeating the entire model architecture search. We compared the RE-epoch detection performance and AHI estimation error (i.e. average bias and limits of agreement) obtained with the HRV-centered method with our proposed method in order to prove the value of the additional features, i.e. sleep stages probability and respiration activity features. The comparison was based on the hold-out test set with the exclusion of low rPPG quality recordings.

The RE-epoch detection performance of the proposed method and the one with the reduced feature set are reported in Table S1. The AHI estimation was characterized by 5.3±23.8 events/h average bias and limits of agreement. The deep learning model selected using only HRV features was the same as the proposed method with the complete feature set. The additional features improved both the RE-epoch detection and AHI estimation performance. For the HRV-centered model, the epochs characterised by limb movements contributed to 38% of the false-positive detection and the specificity, sensitivity and PPV in these epochs were respectively 90%, 34% and 31%.

The addition of respiratory activity and sleep stage probability features to the HRV variability features contributed to increasing the confidence in the RE-epoch detection. A tight connection exists between respiratory activity features and the investigated phenomena, i.e. sleep events affecting the respiratory activity; therefore, we think they played a crucial role in distinguishing RE-epochs from non-RE-epochs and especially those influenced by autonomic activity confounding factors. The increase of sensitivity and PPV with an unchanged specificity obtained with the full feature set in comparison with the HRV-centered set supported our hypothesis and it translated in a lower AHI underestimation and narrower limits of agreements. The lower susceptibility to limb movements of the HRV-centered method was exchanged for a lower true-positive detection rate. This trade-off generated a method too cautious in assigning the positive class and, therefore, more prone to AHI underestimation.

Features	Cohen's kappa	Accuracy [%]	Sensitivity [%]	Specificity [%]	PPV [%]	PR AUC	ROC AUC
HRV + Respiratory activity + Accelerometer + Context	0.37	86	39	94	51	0.47	0.82
HRV + Accelerometer	0.31	85	31	94	49	0.42	0.79

Table S1. Comparison of the RE-epoch detection performance on the hold-out set without the low rPPG quality recordings exclusion and for the complete and reduced features sets. The performance is for the overall number of epochs contributing to the AHI (i.e. not Wake and epochs with less than 80% undefined features).

Deep learning model

Deep learning models training

The general architecture of a deep learning model for supervised learning consists of a series of stacked layers, with layer-dependent characteristics (hyper-parameters), which connect the input features with the desired output, i.e. epoch-by-epoch probability for positive class. We explored several possible combinations of blocks of layers and hyper-parameters, summarized in Table S2. The work of Radha et al.[68] inspired us to include LSTM and dense layers in our blocks. The choice of using convolution was based on the capability of this type of layers to extract meaningful information from multidimensional

time-series with lower risk of over-fitting than other type of layers, such as recurrent and dense layers. We performed this exploration from the simplest to the most complex structures and empirically adjusted our exploration based on the AHI estimation results obtained on the training and validation sets, i.e. blindly from the hold-out test set. As an example, only a few long-short term memory (LSTM) -based models were explored because we noticed that they were usually performing worse than other architectures during our initial explorations.

**Block type**	**Structure**	**Hyper-parameters**
Stacked dense block	Dense(C)<br>+ Time distributed dropout<br>+ N × [Dense(C/2)<br>+ Time distributed dropout]	C = [128, 64, 32]<br>Dropout rate = [0, 20%]<br>N = [0, 1]<br>Activation = sigmoid
Stacked convolution block (type 0)	N × [Convolution(F, K)<br>+ Batch normalization]	F = [32, 64, 128]<br>K = [3, 5, 7]<br>N = [1, 3, 5]<br>Activation = exponential linear unit
Stacked convolution block (type 1)	N × [Convolution(F, K)<br>+ Batch normalization]<br>+ Gaussian Noise<br>+ N × [Convolution(F, K*)<br>+ Batch normalization]<br>+ Gaussian Noise	F = [32, 64, 128]<br>K = [3, 5, 7]<br>K* = K with dilation rate of 2<br>N = [1, 3, 5]<br>Activation = exponential linear unit<br>Noise standard deviation = [0.01, 0.1]
Stacked convolution block (type 2)	N x [Convolution(F, K)<br>+ Batch normalization)<br>+ Gaussian Noise<br>+ N × [Convolution(F/2, K)<br>+ Batch normalization]<br>+ Gaussian Noise	F = [32, 64, 128]<br>K = [3, 5, 7]<br>N = [1, 3, 5]<br>Activation = exponential linear unit<br>Noise standard deviation = [0.01, 0.1]
Stacked convolution block (type 3)	N × [Convolution(F, K)<br>+ Batch normalization)<br>+ Gaussian Noise<br>+ N × [Convolution(F/2, K)<br>+ Batch normalization]<br>+ Gaussian Noise<br>+ N × [Convolution(F, K*)<br>+ Batch normalization]<br>+ Gaussian Noise	F = [32, 64, 128]<br>K = [3, 5, 7]<br>K* = K with dilation rate of 2<br>N = [1, 3, 5]<br>Activation = exponential linear unit<br>Noise standard deviation = [0.01, 0.1]
Stacked LSTM block	N × [Bidirectional LSTM(C)<br>+ Time distributed dropout]	C = [32, 64]<br>Dropout rate = [30%, 50%]<br>Activation = tanh<br>Recurrent activation = hard sigmoid
Noise block	Gaussian Noise	Noise standard deviation = [0.01, 0.1]
Input drop-out (optional first block)	Time distributed dropout	Dropout rate = [0%, 20%]
Output block (always last block)	Dense(C)<br>+ Time distributed dropout<br>+ Dense(1)	C = [32, 16]<br>Dropout rate = [0%, 30%]<br>Activation = sigmoid

Table S2. Blocks of layers and hyper-parameters used to search for the best RE-epoch detection deep learning model. C = number of neurons, F = number of filters, K = kernel size, N = number of stacked layers. Hyperparameter belonging to all the layers: Kernel constraint = maxnorm(3).

Some examples of model architectures searched (independently by the hyperparameters used):

- Input drop-out → Stacked dense block → Stacked LSTM block → Output block
- Input drop-out → Stacked dense block → Stacked convolution block (type 1) → Output block
- Input drop-out → Noise block → Stacked convolution block (type 3) → Stacked convolution block (type 3) → Stacked convolution block (type 2) → Output block

For each model, we set the maximum number of training iterations to 5000 and used a batch size of 32 overnight recordings. The loss function used was a weighted binary cross-entropy and the optimizer a root mean square propagation optimizer with a learning rate of 0.0001. The training stopped when the validation set loss did not improve at least by 0.0005 for 300 consecutive training iterations, and we selected the model with the lowest validation loss as the final model. The models were been implemented in a Python 3.6 environment using the Keras functions of the Tensorflow library (version 1.14).

The amount of RE-epochs is lower than the amount of non-RE-epochs. To compensate for the class imbalanced, we calculated the loss weight of each epoch as:

$$C_w = \frac{N^{tot.\ epochs}}{N^{RE-epochs} + N^{adj.\ RE-epochs}}, \tag{3}$$

$$loss\ weight(i) = \begin{cases} C_w, & \text{if epoch(i) or epoch(i}\pm\text{1) are RE-epochs} \\ 1, & \text{otherwise} \end{cases} \tag{4}$$

where $N^{RE-epochs}$, $N^{adj.\ RE-epochs}$ and $N^{tot.\ epochs}$ are, respectively, the amount of RE-epochs, the amount of non-RE-epochs adjacent to a RE-epoch and the total amount of epochs. In this manner, we also forced the model to pay attention to the change in class from one epoch to the next. Epochs with more than 80% of undefined features were considered unreliable, and they were assigned loss weight of zero.

The output of each model, i.e. positive class probability, was thresholded to define a positive class detection. The choice of the probability threshold was performed on the training set based on Cohen's kappa between reference and estimated OSA severity[27]).

Deep learning model selection

After training over a thousand different deep learning models to perform RE-epochs detection, we selected the model that performed best in AHI estimation in the validation set. More specifically,

1. We selected the 20 models with unique combinations of blocks, used independently from their hyper-parameters, that had the highest Cohen's kappa[88] between reference and estimated OSA severity;
2. These selected models were retrained eight times by re-randomizing training and validation sets. In this way, we estimated the resilience to changes in training and validation data;
3. We selected the model architecture with the best median Cohen's kappa of OSA severity;
4. For the selected model architecture, we selected the model using the training and validation sets split with the highest Spearman's correlation between the reference and estimated AHI.

We decided to select the model based on the AHI estimation performance instead of the RE-epochs detection performance to avoid possible bias introduced by participants with few respiratory events (i.e. cases when positive class is absent or extremely unbalanced). Even though the OSA severity is an ordinal quantity, we opted for standard Cohen's kappa instead of its weighted version[66] to have a stricter evaluation of each misclassification. The last selection step employed the Spearman's correlation, as opposed to Cohen's kappa, because it can account for the spread within each OSA severity class.

Performance of the selected model architecture

The twenty unique architectures selected in step 1 of the model selection had more than a fair agreement between the reference and the estimated OSA severity in the validation set (minimum OSA severity: Cohen's kappa of 0.37 amongst the 20 selected). Most of these architectures were stacked convolutional blocks spaced by blocks to reduce over-fitting (Gaussian noise layers). The selected model architecture (step 3. of the model selection) had a median and IQR of the OSA severity Cohen's kappa on the re-randomized validation sets equal to 0.36 [0.26 – 0.38].

Influence of rPPG quality on performance

The performance after the exclusion of low-quality recordings showed an increase with respect to the AHI estimation. Therefore, we tested wheter further tightening of the rPPG quality requirements would increase performance. Since the initial low-quality definition was based on multiple parameters derived from the training, we decided to maintain a similar definition for the more tight quality requirements. We calculated the parameters reported in Table 3 also for 20th, 30th, 40th and 50th percentile of the quality of the training set recordings (Table S3).

Increasing the rPPG quality requirements generated an overall increase in performance for RE-epoch detection (Table S4) and AHI estimation (Table S5) at the cost of a decreased amount of recordings available.

rPPG quality metrics	Minimum values of training set quality percentiles 10th	20th	30th	40th	50th
IBI coverage [%]	83	87	90	91	93
Average pulse quality index	0.85	0.90	0.91	0.92	0.93
Median pulse quality index	0.90	0.95	0.96	0.97	0.97
Percentage of pulses with quality index > 0.6 [%]	89	94	95	96	97
25th percentile pulse quality index	0.80	0.85	0.88	0.89	0.91
75th percentile pulse quality index	0.96	0.99	0.99	0.99	0.99

Table S3. Incremental rPPG quality recording exclusion criteria. These metrics were calculated for each 30-seconds epoch and averaged for each recording .

RE-epoch detection performance metric	rPPG quality requirement None	10th	20th	30th	40th	50th
Cohen's kappa	0.36	0.37	0.37	0.38	0.39	**0.41**
Accuracy [%]	85	86	87	88	88	**88**
Sensitivity [%]	38	39	39	41	42	**44**
Specificity [%]	94	94	94	94	94	**94**
PPV [%]	53	51	51	49	51	**53**
PR AUC	0.48	0.47	0.46	0.47	51	**0.51**
ROC AUC	0.80	0.82	0.82	0.83	83	**0.85**
Epochs (RE-epochs) [#]	222039 (32456)	171237 (22305)	122319 (17468)	95933 (12728)	83637 (11105)	62543 (8374)

Table S4. RE-epoch detection performance and the number of remaining RE-epochs when the rPPG quality requirements increase. In bold the best performance values (before rounding).

AHI estimation performance metric	rPPG quality requirement None	10th	20th	30th	40th	50th
Spearman's correlation	0.61	0.67	0.62	0.63	0.64	**0.71**
ICC(2,1)	0.64	0.68	0.68	0.72	0.72	**0.76**
ICC(2,1) 95th CI	0.51 - 0.74	0.57 - 0.76	0.57 - 0.77	0.62 - 0.80	0.62 - 0.81	**0.65 - 0.84**
bias [events/h]	4.7	3.3	3.1	**2.1**	2.2	2.2
1.96 * bias standard deviation [events/h]	23.5	19.9	19.3	17.3	17.3	**16.9**
OSA severity weighted Cohen's kappa	0.46	0.51	0.51	0.54	0.56	**0.60**
Recordings [#]	252	188	157	123	106	78

Table S5. AHI estimation performance and the number of remaining recordings when the rPPG quality requirements increase. In bold the best performance values (before rounding).
